# The role of neuropeptide-Y in nandrolone decanoate-induced attenuation of antidepressant effect of exercise

**DOI:** 10.1371/journal.pone.0178922

**Published:** 2017-06-05

**Authors:** Jovana Joksimovic, Dragica Selakovic, Milovan Matovic, Ivan Zaletel, Nela Puskas, Gvozden Rosic

**Affiliations:** 1 Department of Physiology, Faculty of Medical Sciences, University of Kragujevac, Kragujevac, Serbia; 2 Deparment of Nuclear Medicine, Faculty of Medical Sciences University of Kragujevac, Clinical Centre "Kragujevac", Kragujevac, Serbia; 3 Institute of Histology and Embryology “Aleksandar Đ. Kostić”, School of Medicine, University of Belgrade, Belgrade, Serbia; University of Modena and Reggio Emilia, ITALY

## Abstract

Since the increased prevalence of anabolic androgenic steroids abuse in last few decades is usually accompanied by various exercise protocols, the scope of our study was to evaluate the effects of chronic nandrolone decanoate administration in supraphysiological dose and a prolonged swimming protocol (alone and simultaneously with nandrolone decanoate) on depressive state in male rats. Simultaneously, we investigated the possible alterations in neuropeptide Y (NPY) content in blood and the hippocampus, in order to determine the role of NPY in the modulation of depressive-like behavior.Exercise induced antidepressant effects in tail suspension test (decrease of the total duration of immobility), as well as significant increase in the number of hippocampal NPY-interneurons in CA1 region. Chronic nandrolone decanoate treatment attenuated the beneficial antidepressant effects of exercise as measured by the tail suspension test parameters. Simultaneously, nandrolone decanoate treatment resulted in diminution of NPY content both in blood (decreased serum levels) and in hippocampus (the significant decrease in NPY expression in all three investigated hippocampal regions—CA1, CA2/3 and DG). Our findings indicate that alterations in serum and hippocampal NPY contents may underlie the changes in depressive state in rats. The exercise was beneficial as it exerted antidepressant effect, while chronic nandrolone decanoate treatment resulted in depressive-like behavior. Furthermore, the behavioral indicators of depression showed strong correlations with the serum levels and the hippocampal content of NPY.

## Introduction

Anabolic androgenic steroids (AASs), synthetic derivatives of testosterone, have been used for therapeutical purposes since the middle of the twentieth century. Parallel with the therapeutic use of AASs, top athletes began to abuse these substances as powerful doping agents. Expectedly, there is a growing number of evidence that AASs abuse has been widely spread even among the non-athlete adolescent males [1]. Ever since, the information considering the impact of AASs on the central nervous system has been multiplied [2]. Thus, it has been reported that AASs dependence (following chronic application) may be connected with pathogenesis of numerous psychiatric disorders, such as: mania and hypomania, violent behavior, suicide, anxiety, paranoia and depression [3].

Numerous studies performed on animal models have been carried out in order to allow better insight in AAS-induced behavioral changes. For example, it has been reported that prolonged administration of one of the most frequently misused AASs, nandrolone decanoate (ND), in supraphysiological dose resulted in increased anxiety levels [4] in sedentary male rats. However, the results for AASs effects on depressive-like behavior are still contradictory [5]. While low doses of AAS have been shown to induce antidepressant effect in male rats [6], repeated administration of higher dose of AAS resulted in changes indicative of a depressive state in normal rats, giving the evidence that AASs abuse in humans may cause depression regardless of exposure to stress or other risk factors [7]. Previous reports confirmed beneficial effects of chronic exercise by means of various behavioral manifestations. Chronic exercise protocols produced both anxiolytic and antidepressant behavioral effects in rats [8]. However, results concerning behavioral effects of simultaneous administration of AASs along with exercise protocols are very inconsistent. Probably due to diversity of exercise protocols (e.g. different load, duration, type…), as well as AASs treatment (dose, duration…), different behavioral effects following combined protocols presented in literature are hardly comparable.

There has been a huge effort to reveal neurobiology of depression and anxiety by means of behavioral investigations in animal models. Although stress plays a major role in pathogenesis of mental disorders, specific mechanism underlying behavioral manifestations of depression and anxiety remains unclear. In animal models, neurobehavioral markers concerning depression and anxiety comorbidities are also of great interest [9]. It has been confirmed that numerous brain structures, such as amygdala, prefrontal cortex, and the most frequently–hippocampus, are involved in pathogenesis and behavioral manifestations of depression [10]. Although hippocampus has a well-established role in anxiety [11], there is an increasing array of evidence concerning hippocampal structure alterations in depression disorders, by means of hippocampal volume [12] or dysfunction of hippocampal GABAergic system [13].

Neuropeptide Y (NPY) is the most abundant neuropeptide widespread in different brain regions with an important role in the regulation of basic physiological functions, and may be connected to several psychiatric disorders, including depression and related illnesses [14]. Previous studies showed that NPY-like immunoreactivity was significantly lower in cerebrospinal fluid of depressive patients [15], whereas it was significantly higher in brain tissue following antidepressant [16]. Furthermore, intracerebroventricular injection of NPY exerts antidepressant effects [17], suggesting that NPY has an important role in mechanisms involved in emotional and behavioral response to stress. Also, plasma NPY levels were significantly lower in patients who recently attempted suicide [18]. It has been reported that elevated glucocorticoid levels in blood induced significant decrease of NPY plasma levels in healthy subjects with no effect in depressed patients [18].

The present study was performed in order to evaluate the effects of a prolonged exercise protocol and chronic administration of ND in supraphysiological dose (equal to common doses for AASs abuse in humans) on behavioral alterations that can be attributed to a depressive state. At the same time, the aim of this study was the assessment of possible alterations of NPY content in rat hippocampus and peripheral circulation, in order to estimate the possible role of NPY in modulation of depressive-like behavior following chronic ND and exercise pretreatment.

## Materials and methods

### Ethic statement

This study, including pretreatment procedures, was carried out in strict accordance with the European Directive for welfare of laboratory animals N° 86/609/EEC and principles of Good Laboratory Practice (GLP). The protocol was approved by the by Ethical Committee of the Faculty of Medical Sciences, University of Kragujevac, Serbia.

### Animals

Three months old male Wistar albino rats (weighting between 350–400 g, n = 44) were used in this study. Animals were housed in groups of 3–4 per (polycarbonate) cage, under controlled environmental conditions of temperature (23±1°C) and light (12/12h light/dark cycle). Rats had ad libitum access to food and water. Animals were divided into four groups (11 animals in each group): control group (C group), nandrolone decanoate group (ND group), exercise group (E group) and nandrolone decanoate plus exercise group (ND+E, i.e. combined group).

### Treatment

ND group received ND (20 mg/kg/weekly, dissolved in sterilized olive oil up to 0.5 ml as the total volume, s.c.) once a week for six weeks (DEKA 300, SteroxLab, EU). The applied dose of ND (50–100 fold higher compared to physiological levels of androgens) was defined in order to mimic the doses for heavy human AASs abusers [19]. The rats from exercise group performed swimming protocol (60 minutes per day, 5 consecutive days with 2 days break, for six weeks), in a group of four animals. Exercise was performed in a heated (32 ± 1°C) glass tank (60 x 75 x 100 cm) with water depth of 60 cm. Initially, to all animals to familiarize with water contact, rats were kept in a tank with water depth of 7 cm for 15 minutes daily for one week before the text in order to reduce water-induced stress, without promoting significant physiological alterations related to physical training, expressed through stress biomarkers [20]. The duration of a single swimming trial was set within the range of swimming protocols that induced significant immunohistochemical changes in rat brain [21]. ND+E group received ND (20 mg/kg/weekly, s.c.) and simultaneously performed the same swimming protocol as exercise group for six weeks. Control and exercise groups had received approximately the equal volume of sterilized olive oil in the same manner as ND and ND+E groups received therapy. Subcutaneous injections were administrated to all animals on the first day (at 9 a.m.) after finishing the exercise protocol (day 6 of weekly protocol) in order to avoid the potential complications of parenteral application following water immersion. Rats from control and ND group were placed in water for short time (30 seconds) each day of training protocol, in order to eliminate the effect of stress caused by immersion in water between exercise and non-exercise groups. During the entire duration of the swimming task, the experimenter was present to monitor the rats. All rats were able to swim for whole 60 minutes. At the end of swimming sessions all rats were towel dried and placed in a clean cage.

Two days following six weeks of pretreatment, in order to acclimate, the rats were placed in a testing room for 1–2 h prior to initiation of behavioral testing.

After behavioral testing, approximately at 5 p.m., the rats were anaesthetized by short-term narcosis, induced by intraperitoneal application of ketamine (10 mg/kg) and xylazine (5 mg/kg), and then scarified by decapitation. Brains were carefully removed for further histological analysis, while trunk blood samples were collected for serum hormone assays.

### Tail suspension test

In order to evaluate the level of depressive state, we performed a tail suspension test (TST). TST is based on the assumption that an animal will actively try to escape an aversive (stressful) stimulus [22]. Rats were suspended by the tail attached to the adhesive tape, so that their bodies dangled in the air facing downward, both acoustically and visually isolated. Immobility was considered as a state of the animal with no visible voluntary movement (less than 1 cm) of head, body or limbs for 5 seconds or more. Involuntary swinging was considered as immobility. The tests lasted for six minutes. The test apparatus consisted of metal frame (60 x 60 cm) and circular barrier (25 cm in diameter) with the central opening (1.5 cm in diameter) where the tails were slipped through, 1 cm bellow the position of the adhesive tape on the tail, in order to prevent tail climbing. Behavior of the rats was recorded by a video camera, and records were analyzed in order to determine the following parameters: the latency to the first immobility, the number of immobility episodes, the total duration of immobility and an average duration of an immobility episode (the ratio of total duration of immobility and the number of immobility episodes).

### Serum hormone assays

Blood samples (1.5 ml) were collected, prepared and stored (at -80°C) until serum analysis according to previously described procedure [23]. Serum samples were assessed for cortisol and NPY levels by Elecsys 2010 analyzer using the method of radioimmunoassay (RIA). Standard commercial kits (CORT—CT 2, CisBio Bioassays, Codolet, France and NPY RIA kit, RK-049-03, Phoenix Pharmaceuticals, Inc. Bunrlingame, USA) were used and the cortisol and NPY levels were expressed in ng/ml and pg/ml, respectively. The sensitivity of the assays for cortisol and NPY were 2.39 ng/ml and 69 pg/ml, respectively. Inter- and intra-assay coefficients of variance were 5.7 and 5.3%, respectively, for cortisol, 12 and 5%, respectively, for NPY, according to manufacturers’ specifications.

### Immunohistochemistry

After sacrificing the rats (S1 File), brains were carefully removed from the skull, fixated in 4% formaldehyde solution in phosphate buffer and embedded in paraffin (S1 Table). Coronal brain sections, 5 μm thick, were dewaxed, rehydrated and treated with citrate buffer (pH 6.0) in a microwave for antigen retrieval. Endogenous peroxidase activity was blocked with 3% H_2_O_2_, and nonspecific labeling was blocked by commercial protein block (Novocastra, UK). Slices were incubated in rabbit polyclonal anti-NPY (1:250, AbD Serotec) overnight at room temperature. The immunohistochemistry procedure on formalin-fixed paraffin-embedded tissue was done according to the methodology described by Nowak and coworkers [24] with incubation in primary antibody overnight, with slight modification. Labeling was performed using biotin-conjugated secondary antibodies, followed by streptavidin-HRP, and visualization was done with 3,3’-diaminobenzidine (DAB) chromogen (all components from Peroxidase Detection System RE 7120-K, Novocastra, UK). Finally, sections were counterstained with Mayer’s hematoxylin and covered. The staining specificity was checked by omitting the primary antiserum. No immunoreactivity was detected in these sections. Image capturing of NPY stained hippocampal slices was done on Leica DM4000 B LED microscope with digital camera Leica DFC295 and by using Leica Application Suite (LAS, v4.4.0) software system. The surface area of each of the hippocampal regions (CA1, CA2/3, DG) in the chosen sections was measured by the above-mentioned software system and the number of NPY immunoreactive cells was counted in each of those areas (S2 Table), after which the number of counted immunoreactive neurons was expressed per 1 mm^2^ of investigated region in order to standardize the number of counted cells. The counting was always done on the dorsal hippocampus (level of section was 3.80 mm caudal to the bregma, according to Paxinos and Watson stereotaxic atlas [25]), on one hippocampal section per animal, and on all animals from control and experimental groups (11 rats per group).

### Statistical analysis

The data presented herein were expressed as the means ± S.E.M. Parameters obtained in behavioral tests were initially submitted to Levene's test for homogeneity of variance and to Shapiro-Wilk test of normality. Comparisons between groups were performed using One-way ANOVA, followed by Fisher's least significant difference (LSD) multiple comparisons test, for behavioral tests parameters and serum hormone levels, and with Bonferroni testing for morphological analysis. Simple linear regression analyses were performed to analyze relationships between parameters obtained in behavioral tests and histological data. Significance was determined at *p* < 0.05 for all tests. Statistically analysis was performed with SPSS version 20.0 statistical package (IBM SPSS Statistics 20).

## Results

### Tail suspension test

**Fig 1:**
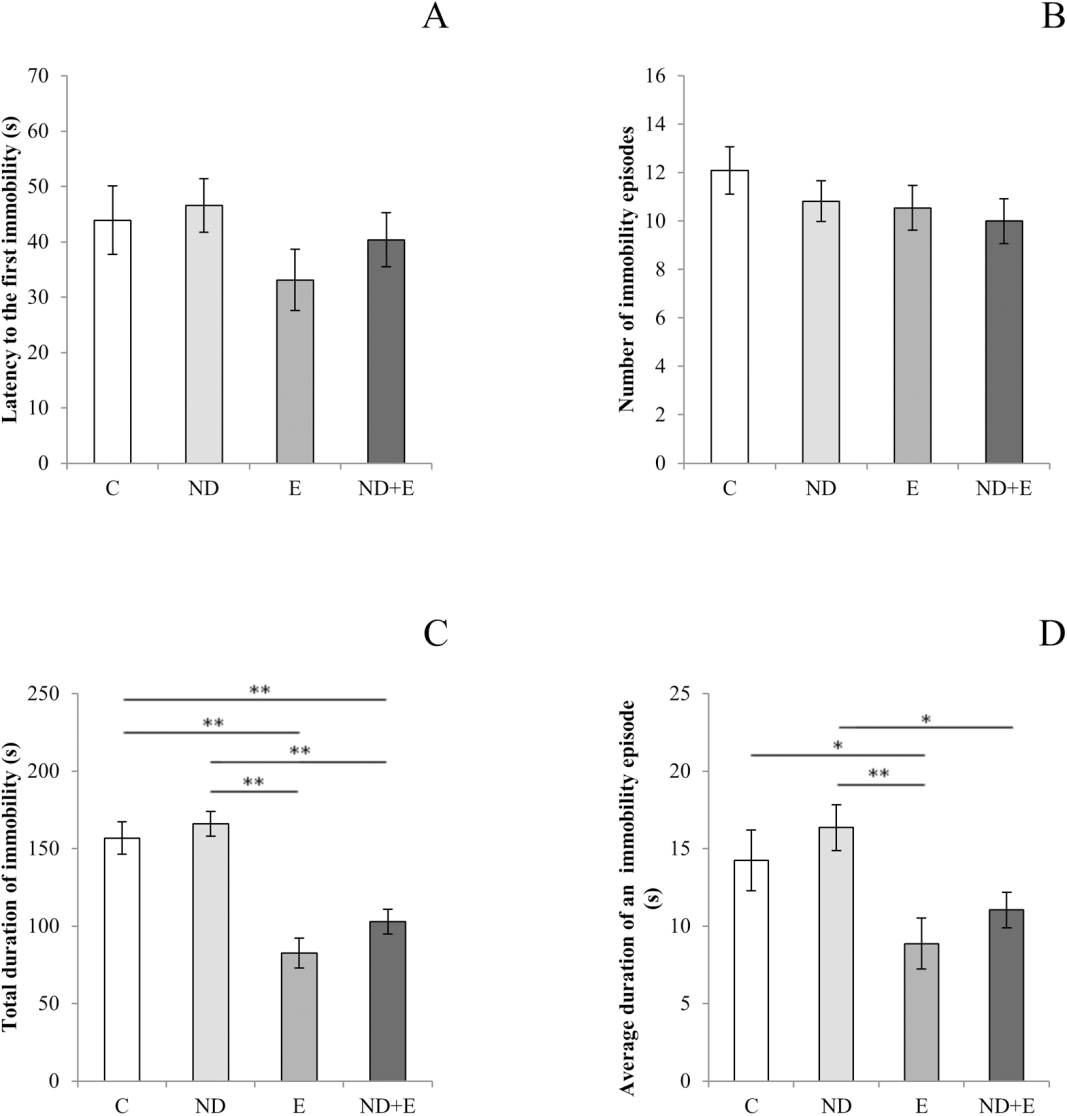
Parameters calculated from the tail suspension test. A–Latency to first immobility; B–Number of immobility episodes; C–Total duration of immobility; D–Average duratation of immobility episode (C—control group, ND–nandrolone decanoate group, E–exercise group, ND+E–nandrolone plus exercise group). Bars represent means ± SEM, n = 11. * denotes a significant difference *p* < 0.05, ** denotes a significant difference *p* < 0.01.

There was no difference among groups in the latency to the first immobility (Fig 1A), as well as in the number of immobility episodes (Fig 1B) in TST (S2 File). However, two other parameters obtained in TST, the total duration of immobility and the average duration of an immobility episode, showed statistical significance among groups (F = 20.403 and 4.391, respectively, df = 3, p<0.01).The total duration of immobility was significantly decreased in the exercise group compared to the control and ND groups. Also, chronic ND administration at supraphysiological dose resulted in a significant increase in total duration of immobility when compared to the combined group (Fig 1C). Simultaneous application of ND along with exercise produced significant decrease in total duration of immobility compared to the control group. Furthermore, the average duration of immobility episode was significantly higher in ND group compared to both exercise and combined groups (Fig 1D). The exercise protocol resulted in significant decrease in the average duration of an immobility episode compared to control.

### Serum NPY and cortisol levels

As shown in Table 1, the chronic ND administration induced significant decrease in NPY serum level (S2 File) when compared to control, exercise and combined (ND+E) group (F = 19.589, df = 3, p<0.01). Although exercise protocol resulted in enhanced NPY serum levels that was not significant compared to control, the exercise-induced serum NPY elevation was significant compared to ND, as well as to combined group (p<0.01). However, serum cortisol levels were not affected following any applied protocol in this study (F = 0.248).

**Table 1:** Serum levels of cortisol and NPY.

Group Hormone	C	ND	E	ND+E
Cortisol (ng/ml)	74.34 ± 4.01	67.08 ± 10.43	65.24 ± 7.43	71.16 ± 9.49
NPY (pg/ml)	203.59 ± 14.69	55.49 ± 10.30 ^a^^**^ ^b^^**^ ^c^^**^	236.76 ± 26.38 ^d^^**^	165.98 ± 15.84

Values are expressed as mean ± SEM (n = 8). C—control group, ND—nandrolone decanoate group, E—exercise group, ND+E—nandrolone decanoate plus exercise group.

^a^ C vs. ND

^b^ ND vs. E

^c^ ND vs. ND+E

^d^ E vs. ND+E

^**^ denotes a significant difference *p* < 0.01.

### Immunohistochemistry

**Fig 2:**
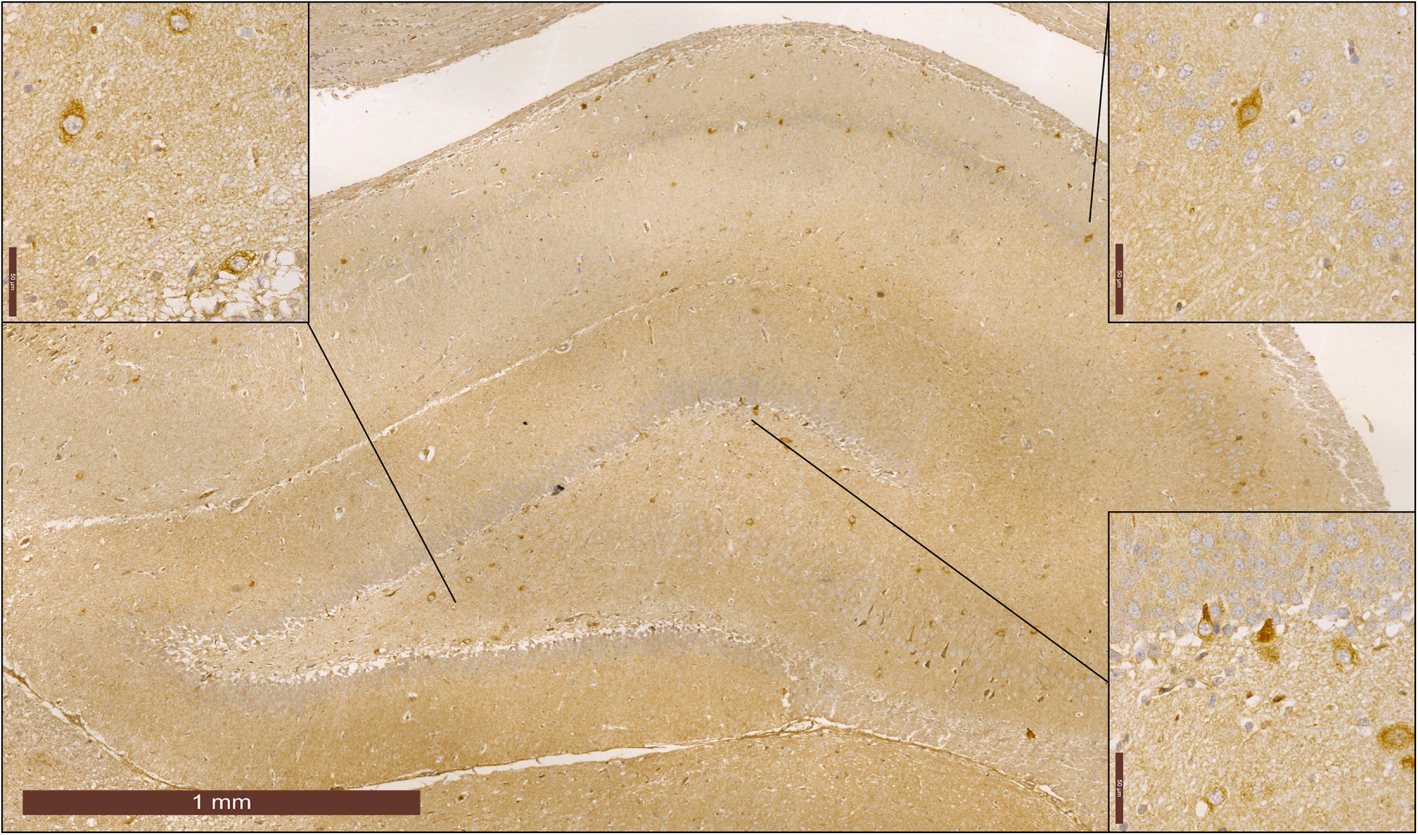
Immunohistochemical expression of NPY positive interneurons in the rat hippocampus from the control group.

**Fig 3:**
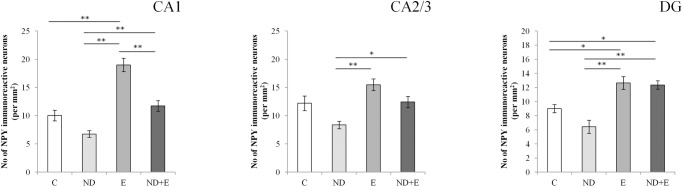
Number of NPY immunoreactive neurons per 1mm^2^ in each of the investigated hippocampal regions (CA1, CA2/3 and DG). C—control group, ND–nandrolone decanoate group, E–exercise group, ND+E—nandrolone decanoate plus exercise group. Bars represent means ± SEM, n = 11. * denotes a significant difference *p* < 0.05, ** denotes significant difference *p* < 0.01.

For all investigated groups, NPY immuoreactive neurons in hippocampus (S2 File) were predominantly located in pyramidal cell layer of CA1 and CA2/3 regions, whereas in DG NPY immuoreactive neurons were expressed mostly in the hilus (Fig 2). Statistical analysis of NPY interneurons expression in hippocampus (Fig 3) showed statistical significance between groups in all three regions of the hippocampus (df = 3; CA1: F = 30.163, CA2-3: F = 8.114, DG: F = 14.383, p<0.0001). Six-week swimming protocol resulted in a significant increase in the number of NPY immunoreactive neurons in CA1 and DG compared to control, and in all three investigated hippocampal regions compared to ND group, while chronic ND treatment had no significant influence on the number of NPY immunoreactive neurons compared to the control group. Chronic exercise protocol along with ND treatment significantly increased number of NPY interneurons compared to the control (in DG) and ND group (in all three hippocampal regions).

**Fig 4:**
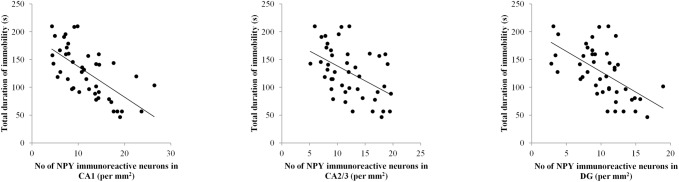
Relationship between the number of NPY immunoreactive neurons in different regions of hippocampus and the total duration of immobility in TST for all investigated groups (n = 44). Simple regression analysis indicated that the number of NPY neurons in all three investigated hippocampal regions: CA1 (Pearson's r = 0.652, p = 0.000001), CA2/3 (Pearson's r = 0.486, p = 0.001) and DG (Pearson's r = 0.576, p = 0.00004) was negatively correlated with the duration of immobility in TST.

**Fig 5:**
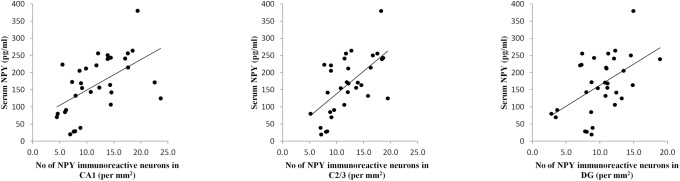
Relationship between the number NPY immunoreactive neurons in different regions of hippocampus and the serum NPY levels for all investigated groups (n = 32). Simple regression analysis indicated that the number of NPY neurons in all three investigated hippocampal regions: CA1 (Pearson's r = 0.54, p = 0.001), CA2/3 (Pearson's r = 0.61, p = 0.0001) and DG (Pearson's r = 0.50, p = 0.003) was positively correlated with the serum NPY levels.

**Fig 6:**
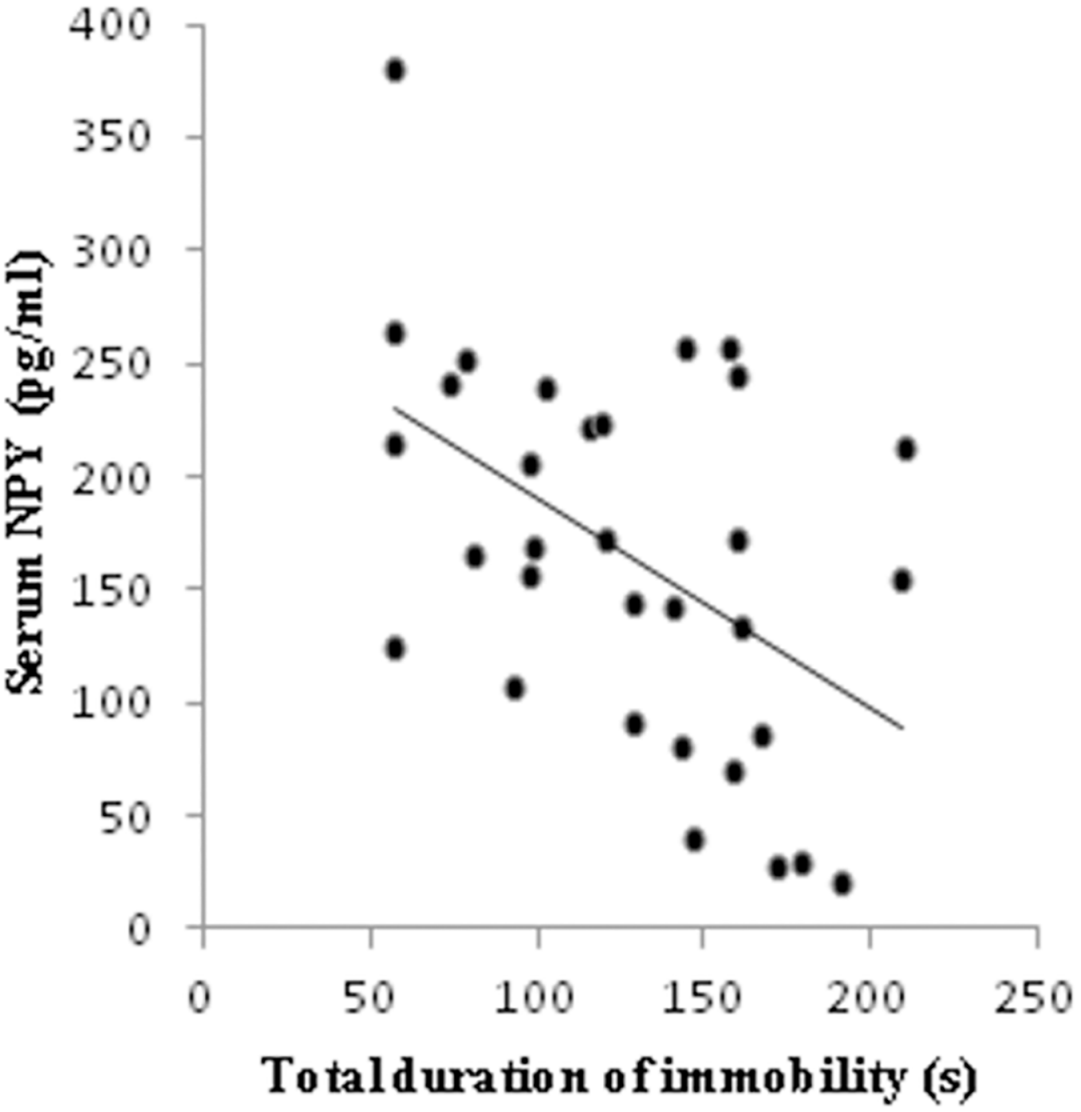
Relationship between the total duration of immobility in TST and the serum NPY levels for all investigated groups (n = 32). Simple regression analysis indicated that the serum NPY levels (Pearson's r = 0.48, p = 0.004) was negatively correlated with the duration of immobility in TST.

Simple regression analysis revealed that the number of NPY immunoreactive neurons in all three investigated hippocampal regions (CA1, CA2/3 and DG) was significantly negatively correlated (Fig 4) with the total duration of immobility in TST (Pearson's r = 0.652, p = 0.000001; r = 0.486, p = 0.001 and r = 0.576, p = 0.00004, respectively). The same method of analysis also confirmed significant (positive) correlation (Fig 5) between the number of NPY immunoreactive neurons in named hippocampal regions and the serum NPY levels (CA1—r = 0.54, p = 0.001, CA2/3—r = 0.61, p = 0.0001 and DG—r = 0.50, p = 0.003). Finally, as shown in Fig 6, the serum NPY levels significantly negatively correlated with the total duration of immobility in TST (r = 0.48, p = 0.004).

## Discussion

The present study was performed in order to evaluate the effects of supraphysiological doses of ND and exercise on depressive state levels, as well as to estimate the possible role of two different pools of NPY (peripheral—in blood and central—hippocampal) and cortisol in modulation of this behavioral pattern in rats. Results of this study showed that chronic exercise protocol, such as performed in this study, induced antidepressant effect by means of TST parameters. TST was chosen as the test for estimation of depression state levels because, beside the forced swim test, it represents traditional model for estimation of depression-like behavior in rodents. However, we found the TST as more suitable test for this study, since we intended to overcome the expected differences in performance between the groups of animals that could possibly be more accommodated to water environment (exercise and combined group) and sedentary groups (control and ND group). Our results are in accordance with previous reports concerning antidepressant effect of exercise in rats [26]. The most prominent manifestation of exercise-induced antidepressant effect was observed by means of total duration of immobility (decrease by 50%, p<0.001, compared to control), the parameter that is commonly considered as the key indicator for antidepressant action in similar trials [27]. However, chronic ND treatment at supraphysiological dose produced opposite effects on depressive state level in TST. Although with no significant change compared to the control values observed in the sedentary group, ND administration resulted in significant increase of the total duration of immobility and in average duration of an immobility episode when compared to the exercise group. Those prodepressant effects of ND were confirmed following simultaneous ND administration along with an exercise protocol, where ND attenuated the beneficial (antidepressant) effects of exercise by prolonging the immobility time and average duration of an immobility episode (Fig 1). Although, AASs in physiological doses do not affect or even show beneficial effects [28], our results are consistent with previous reports showing that supraphysiological doses of AASs may contribute to depression genesis [29], where higher doses resulted in more severe clinical manifestations [30]. Similar effects of supraphysiological doses of ND on depression-like behavior were observed in rats [5, 31].

Since in numerous reports cortisol has been attributed to be involved in various mood disorders, including alterations in depressive state [32], we estimated the effect of all applied protocols on cortisol levels in blood and its connections to behavioral changes indicative for depression. Determination of serum cortisol levels was performed in order to estimate the alterations of glucorticoid levels depending on applied protocol. Considering the fact that cortisol is released in parallel with corticosterone [23], which is the most prominent glucocorticoid in rodents [33], determination of cortisol levels was performed in order to evaluate the effects of applied protocols on this antistress hormone levels in blood. Despite of pronounced behavioral alterations in TST, none of the applied protocols in this study affected serum cortisol levels. The effects of various exercise protocols on glucocorticoid levels were investigated in numerous studies [34, 35]. The blood samples used for the estimation of serum cortisol levels were collected two days after completing the chronic exercise protocol to maintain the design established in this investigation—5 days of swimming / 2 days break. That algorithm also allows the estimation of hormonal status at the time the behavioral testing is performed (commonly a few days after completing the chronic training protocols in order to minimize the acute effects of exercise on behavioral patterns).It has been reported that significant exercise-induced elevation of glucocorticoid levels in rodents was observed during and immediately after acute exposure to heavy exercise [20], but with no change in glucocorticoid levels 24 hours following the completion of chronic exercise protocol [36].The results regarding the effects of AASs on serum glucocorticoid levels are very conflicting. It has been shown that cortisol/corticosterone levels were increased following boldenone administration, while ND decreases serum levels of cortisol depending on AAS treatment duration in rabbits [37]. However, the lack of significant influence of AAS, as observed in this study, is in line with previous report for testosterone propionate action in mice [38]. Consistently, the serum cortisol levels observed following simultaneous administration of ND and exercise in this study remained unchanged.

In contrary to cortisol in blood, serum NPY levels were significantly affected by protocols performed in this study. The increase of serum NPY following exercise protocol in this study was not significant compared to control values. This is in line with previous report for the effect of prolonged exercise of moderate intensity (as performed in this study) that had no influence on plasma NPY levels, whereas high intensity exercise resulted in significant elevation of plasma NPY in rodents [39]. The similar effects of exercise on NPY concentration in blood was previously observed in humans one hour after completing the intense exercise protocol [40]. The proposed explanation for the acute and short term increase in NPY levels during extreme exercise was found in increased catecholamine metabolism, both in brain and peripheral tissues, accompanied with simultaneous increase of NPY release [41]. To our knowledge, there is no literature data addressing the effects of AAS on serum NPY levels in humans and experimental animal model.

Interestingly, it has been reported that there is significant difference in NPY levels between peripheral and pituitary portal circulation [42]. Three fold higher concentration of NPY in portal blood portion was explained by a significantly augmented metabolism of NPY in certain brain regions. Therefore, we performed the immunohistochemical analysis for NPY expression in the hippocampus, as this neuropeptide was found to be involved in pathogenesis of various mood disorders, including alterations in depressive state levels [43]. Expression pattern of NPY immunoreactive neurons in the hippocampus in control group is somewhat similar to those described in available literature [44, 45], but total number per mm^2^ is less due to the thickness of paraffin sections used in this case.

The swimming protocol performed in this study resulted in significant increase in the number of NPY neurons, especially in CA1 (90%) and DG (40%) regions compared to control. The observed increase in the number of NPY immunoreactive neurons was significant even following ND treatment along with the exercise protocol in DG, compared to control. Those findings correlate with a previous report investigating exercise-induced increase in rat hippocampal NPY mRNA, which appeared simultaneously with the increase in BDNF and VGF [46]. Unlike the beneficial effect of exercise on hippocampal NPY content, chronic administration of ND resulted in lower NPY expression in hippocampus. Although the decrease in the number of NPY neurons was not significant compared to the control group, it was markedly reduced as compared to the exercise groups, with or without simultaneous ND treatment. ND-induced decline of NPY content was observed in all three investigated regions—CA1, CA2/3 and DG (40–65%, 35–45%, 50%, respectively). Our results for ND- induced alteration of NPY expression in rat hippocampus correlate with the report that ND decreased the number of newly born neurons in DG cell culture of male rats [47]. Moreover, inhibition of NPY circuit induced by a different type of AAS (17a-methyltestosterone) has also been reported in ventromedial nuclei and BNST [48]. Furthermore, good insight in interconnection between the alterations in emotional processing (expressed as depression, anxiety or the clinical phenotype of anxious depression) and single nucleotide polymorphism (SNP rs16147) in the NPY gene was found using fMRI in amygdala [49]. The role of alterations in NPY genotype has been reported in patients with depressive disorders following childhood emotional maltreatment [50]. Additional information considering the role of NPY in depressive disorders could be found in reduction of NPY mRNA in hippocampus [51], amygdala [49] and prefrontal cortex [52].Antidepressant effect of chronic exercise as measured by the TST in this study may be connected to changes in hippocampal NPY content [53]. Although the effect of AASs administration on hippocampal morphological alterations (that may be connected to its prodepressant action) has not been extensively evaluated yet, it is known that AASs induce changes indicative of depressive state in rats [7]. This prodepressant effect of supraphysiological doses of AASs was observed in rats that were exposed to stress, as well as in naive animals,and was explained by the alterations in hippocampal BDNF [7]. In addition, study performed on animal model of depression (Flinders sensitive rat line—FSL) showed lowered hippocampal NPY-like immunoreactivity [54]. Also, it has been reported that FSL rats expressed reduction in NPY mRNA in nucleus accumbens and CA regions of hippocampus, as well as the reduction in NPY Y1 receptors mRNA in deferent cortical regions and dentate gyrus [55]. The results obtained by using this animal model suggest that hippocampal Npy’s epigenetic status could be differently affected by external stimuli, such as physical performance [56]. However, prodepressant effect of chronic ND treatment measured by TST does not correlate with our previous report [57], where four week treatment with the same dose of ND resulted in an antidepressant effect in TST. A possible explanation for observed differences, considering the effect of ND on depressive state, could be due to the longer ND treatment applied in this study. Also, since the changes in depressive state usually do not appear alone, and the fact that symptoms of anxiety and depression often coexists (″anxiety-depression spectrum″, [51]), various mood disorders are likely to influence stress-responsive reactions under certain circumstances [58]. Taking into account that NPY modulates both anxiety and depression through the same receptor type—Y1R [59], it seems possible that changes in total hippocampal NPY content may influence various behavioral patterns. Since six week treatment with ND dose applied in this study decreased the expression of NPY immunoreactive neurons in the hippocampus, pronounced antidepressant effect of ND obtained in our previous study may be explained as a consequence of increased anxiety (as the early manifestation of decreased hippocampal NPY). On the other hand, the chronic ND treatment applied in this study resulted in prodepressant effect that could be considered as the late manifestation of AASs action in hippocampus. Obviously, according to the results of this study, mood disorders (anxiety and depression) do not appear simultaneously following ND-induced decrease of hippocampal NPY content. Based on the results reported in the current study, it seems possible that previously reported anxiogenic effect of AASs [57] is sufficient to mimic initially moderate prodepressant effect of AASs abuse, so that clear prodepressant effect can be observed only following long-lasting trials, such as the one performed in this study. Aside of differences in applied protocols (e. g. duration, dose), initial hormonal status (sex, age, gonadectomy) and additional protocols (exercise, sedentary), it seems that time-dependent interference between various behavioral patterns may be the reason for inconsistency in interpretation of AASs influence on depressive state.

Since the total duration of immobility is commonly considered as the most reliable indicator of depressive state in TST, we estimated the relationship between this parameter and the number of NPY immunoreactive neurons in different hippocampal regions (Fig 4). Our results showed strong inverse correlation between depression state (expressed by means of immobility time in TST) and NPY content in CA1, CA2/3 and DG. These data suggest that all three investigated hippocampal regions may be involved in control of depressive state level. Furthermore, as shown in Fig 5, the number of NPY immunoreactive neurons in those specific hippocampal areas also strongly and positively correlated with the serum NPY levels. These correlations may be considered as the proof for the obvious connection between central and peripheral NPY pools. Finally, the NPY content in blood also showed a very strong correlation with the major manifestation od depressive state (total immobility time) observed in TST (Fig 6). Therefore, the results of this study may lead to the conclusion that determination of NPY levels in blood, could be considered as potentially useful marker for estimation of depressive-like behavior in rodents.

## Conclusion

Our findings indicate that alterations in both blood and hippocampal NPY contents may underlie the changes in depressive state in rats. Exercise showed beneficial effects by exerting antidepressant properties, while chronic treatment with ND resulted in depressive-like behavior. Since the serum NPY levels show strong correlation with morphological (number of NPY hippocampal interneurons) and functional (behavioral alterations) appearance of depression, NPY level in blood may be taken into consideration as a potentially useful marker for the evaluation of depressive state levels in rodents.

## Supporting information

S1 File Immunohistochemical protocols. (DOCX)

S2 File Raw data. (XLSX)

S1 Table Overview of literature concerning IHC-P for NPY (or some other antigens) in paraffin-embedded brain tissue sections. (DOCX)

S2 Table Overview of different methodological approaches for counting the number of cells (or nuclei) per certain square area. (DOCX)

## Abbreviations

ND: nandrolone decanoate
AAS: anabolic androgenic steroid
TST: tail suspension test
NPY: Neuropeptide-Y
