# Supplementary material for: The role of neuropeptide-Y in nandrolone decanoate-induced attenuation of antidepressant effect of exercise
Source: PLoS One. 2017 Jun 5;12(6):e0178922. doi: 10.1371/journal.pone.0178922 (PMC5459494; doi:10.1371/journal.pone.0178922)
Supplement: S1 File — (DOCX) [file pone.0178922.s001.docx]

**S2 File. Immunohistochemical protocols**

Tissue fixation and sectioning

After sacrificing the rats their brains were carefully removed from the skull and fixated in 4% formaldehyde solution in phosphate buffer (PBS). Once fixed, the brains were dehydrated by immersing the tissue in a series of ethanol solutions (70%, 80%, 96%, Zorka Pharma, Šabac, Serbia) until absolute alcohol (100%, Zorka Pharma, Serbia). The brains were then immersed in a clearing agent (Xylene, Zorka Pharma, Serbia), after which the brains were embedded with paraffin wax (Bio-plast extra, Bio-optica, Italy). Coronal brain sections, 5 µm thick, were cut using a microtome (Leica RM2235, Leica Microsystems, Germany), transferred to water bath (Leica, HI1210, Leica Biosystems, Germany) and then mounted to glass slides (Superfrost™ Ultra Plus Adhesion Slides, Thermo Scientific, USA).

Immunohistochemical staining

The slides were first deparaffinized and rehydrated by following procedure:

1. Xylene: 3 x 5 minutes

2. 100% (absolute) ethanol: 2 x 5 minutes

3. 96% ethanol: 5 minutes

4. 70 % ethanol: 5 minutes

5. Distilled water: 5 minutes

Due to the formation of methylene bridges during fixation procedure, which can mask the antigenic sites, the slides were treated with sodium citrate buffer (pH 6.0) in a microwave (800W, 21 minutes) to achieve antigen retrieval. The immunostaining procedure was then done by using the commercially available kit (Peroxidase Detection System RE 7120-K, Novocastra, Leica Biosystems, UK) by following procedure:

1. Neutralization of endogenous peroxidase activity (Peroxidase Block RE7101, Novocastra - 5 minutes)

2. Washing in PBS (3 x 5 minutes)

3. Incubation with commercial protein block in order to prevent nonspecific labeling (Protein Block RE7102, Novocastra - 5 minutes)

4. Washing in PBS (3 x 5 minutes)

5. Incubation of slices in rabbit polyclonal anti-NPY (1:250, AbD Serotec) overnight at room temperature

6. Washing in PBS (3 x 5 minutes)

7. Labeling was performed using commercial biotin-conjugated secondary antibody (Biotinylated Secondary Antibody RE7103, Novocastra - 30 minutes)

8. Washing in PBS (3 x 5 minutes)

9. Incubation with streptavidin-HRP (Streptavidin-HRP RE7104, Novocastra - 30 minutes)

10. Washing in PBS (3 x 5 minutes)

11. Visualization with 3,3’-diaminobenzidine (DAB) solution (DAB Chromogen RE7105 and DAB Substrate Buffer RE7106, Novocastra – 5 minutes)

12. Rinsing in distilled water (2 x 5 minutes)

13. Counterstaining with Mayer’s hematoxylin (5 minutes)

14. Rinsing in water (5 minutes).

The slides were then dehydrated, cleared and cover slipped by following procedure:

1. 70 % ethanol: 5 minutes

2. 96% ethanol: 5 minutes

3. 100% (absolute) ethanol: 2 x 5 minutes

4. Xylene: 3 x 5 minutes

5. Mounted with DPX (Sigma-Aldrich, USA) and coverslipped
