# Supplementary material for: The role of neuropeptide-Y in nandrolone decanoate-induced attenuation of antidepressant effect of exercise
Source: PLoS One. 2017 Jun 5;12(6):e0178922. doi: 10.1371/journal.pone.0178922 (PMC5459494; doi:10.1371/journal.pone.0178922)
Supplement: S1 Table — (DOCX) [file pone.0178922.s003.docx]

| **Author** | **Journal** | **Fixation** |
| --- | --- | --- |
| *Cerf et al., 2010* | International Journal of Developmental Neuroscience | Formalin-fixed Wistar rat brains processed for wax (paraffin) embedding |
| *Rafalo et al., 2017* | Prog Neuropsychopharmacol Biol Psychiatry | Fixation in 4% neutral formaldehyde (10% formalin) and embedded in paraffin |
| *Yang et al., 2014* | Brain Behavior and Immunity | Fixed in 4% paraformaldehyde embedded in paraffin |
| *Nowak et al.,2010* | Pharmacological Reports | Fixation in 4% neutral formaldehyde (10% formalin) and embedded in paraffin |
| *Yang et al., 2012* | Journal of Veterinary Science | Fixed in 4% paraformaldehyde and embedded in paraffin |
| *Antonopoulos et al., 1992* | Neuroscience Letters | Perfusion with Bouin’s fluid and embedded in paraffin wax; non-commercial NPY antiserum was applied |
| *Zonis et al., 2013* | Hippocampus | Fixed in 4% paraformaldehyde embedded in paraffin |
| *Zadrozna et., al 2011* | Pharmacological Reports | Fixation in 4% neutral formaldehyde (10% formalin) and embedded in paraffin |
| *Pirone et al., 2014* | Frontiers in Systems Neuroscience | Fixation in 4% neutral formaldehyde (10% formalin) and embedded in paraffin |

**S1 Table. Overview of literature concerning IHC-P for NPY (or some other antigens) in paraffin-embedded brain tissue sections**
