# Supplementary material for: The role of neuropeptide-Y in nandrolone decanoate-induced attenuation of antidepressant effect of exercise
Source: PLoS One. 2017 Jun 5;12(6):e0178922. doi: 10.1371/journal.pone.0178922 (PMC5459494; doi:10.1371/journal.pone.0178922)
Supplement: S2 Table — (DOCX) [file pone.0178922.s004.docx]

| **Author** | **Journal** | **Counting method** |
| --- | --- | --- |
| *Plescia et al., 2014* | Front Psychiatry | N^o^ NPY in different sub-regions of HC/ of 12 sections for each experimental condition |
| *Kirby et al., 2013* | eLife | N^o^ of Ki67 orBrdU/mm^2^ |
| *Yang et al., 2012* | J Vet Sci | N^o^DCX/DG 2.12mm from bregma |
| *Tzeng et al., 2013* | J Biomed Sci | N^o^/250x250 µm^2^ |
| *Meng et al., 2011* | Eur J Pharmacol | BDNF/ mm^2^ of subfileds of HC and GD |
| *Xiao and Jordan, 2002* | Horm and Behav | N^o^ AR positive cells in a 133x133 µm square area in one section per animal approximately 4.30 mm from bregma |
| *Chun Yan et al., 2013* | Lab Anim Res | N^o^ Ki67 or DCX or BrdU/ section, |
| *Filipovic et al., 2013* | Neuroscience | N^o^ PV / section in CA1, CA3 or GD, |
| *Chai et al., 2016* | Neural Regen Res | N^o^DCX/hemisphere |
| *Chai et al., 2016* | Neural Regen Res | N^o^NeuN in DG/mm |
| *Nowak et al.,2010* | Pharmacological Reports | Mean percentage of images with increasing frequencies of positive cells per image: 0, 1-2, 3-4, 5-6, up to 7 |
| *Matsuda et al., 2012* | J Neurosci | N^o^Ki67/mm^2^DG |

**S2 Table. Overview of different methodological approaches for counting the number of cells (or nuclei) per certain square area**
